# Deleterious *AGXT* Missense Variant Associated with Type 1 Primary Hyperoxaluria (PH1) in Zwartbles Sheep

**DOI:** 10.3390/genes11101147

**Published:** 2020-09-29

**Authors:** Anna Letko, Reinie Dijkman, Ben Strugnell, Irene M. Häfliger, Julia M. Paris, Katrina Henderson, Tim Geraghty, Hannah Orr, Sandra Scholes, Cord Drögemüller

**Affiliations:** 1 Institute of Genetics, Vetsuisse Faculty, University of Bern, 3012 Bern, Switzerland; anna.letko@vetsuisse.unibe.ch (A.L.); irene.haefliger@vetsuisse.unibe.ch (I.M.H.); julia.paris@vetsuisse.unibe.ch (J.M.P.); 2 Royal GD, Postbus 9, 7400 AA Deventer, The Netherlands; r.dijkman2@gddiergezondheid.nl; 3 Farm Post Mortems Ltd., Hamsterley, Bishop Auckland, County Durham DL13 3QF, UK; ben@farmpostmortems.co.uk; 4 SRUC Consulting Veterinary Services, Pentlands Science Park, Bush Estate Loan, Penicuik, Midlothian EH26 0PZ, UK; katrina.henderson@sruc.ac.uk (K.H.); timothy.geraghty@sruc.ac.uk (T.G.); hannah.orr@sruc.ac.uk (H.O.); sandra.scholes@sruc.ac.uk (S.S.)

**Keywords:** *Ovis aries*, oxalate nephropathy, whole-genome sequencing, metabolic disease, precision medicine, genetic test

## Abstract

Severe oxalate nephropathy has been previously reported in sheep and is mostly associated with excessive oxalate in the diet. However, a rare native Dutch breed (Zwartbles) seems to be predisposed to an inherited juvenile form of primary hyperoxaluria and no causative genetic variant has been described so far. This study aims to characterize the phenotype and genetic etiology of the inherited metabolic disease observed in several purebred Zwartbles sheep. Affected animals present with a wide range of clinical signs including condition loss, inappetence, malaise, and, occasionally, respiratory signs, as well as an apparent sudden unexpected death. Histopathology revealed widespread oxalate crystal deposition in kidneys of the cases. Whole-genome sequencing of two affected sheep identified a missense variant in the ovine *AGXT* gene (c.584G>A; p.Cys195Tyr). Variants in *AGXT* are known to cause type I primary hyperoxaluria in dogs and humans. Herein, we present evidence that the observed clinicopathological phenotype can be described as a form of ovine type I primary hyperoxaluria. This disorder is explained by a breed-specific recessively inherited pathogenic *AGXT* variant. Genetic testing enables selection against this fatal disorder in Zwartbles sheep as well as more precise diagnosis in animals with similar clinical phenotype. Our results have been incorporated in the Online Mendelian Inheritance in Animals (OMIA) database (OMIA 001672-9940).

## 1. Introduction

Primary hyperoxaluria is a rare autosomal recessive metabolic disease that leads to an accumulation of calcium oxalate in various tissues that finally result in renal failure [1]. In human patients, three types of primary hyperoxaluria are known and caused by homozygous or compound heterozygous variants in three different genes [1]. Variants in the gene encoding alanine-glyoxylate aminotransferase (*AGXT*) are responsible for type I primary hyperoxaluria (PH1; OMIM 259900), type II (PH2; OMIM 260000) is caused by variants in the glyoxylate reductase/hydroxypyruvate reductase gene (*GRHPR*), and type III (PH3; OMIM 613616) is caused by variants in the mitochondrial dihydrodipicolinate synthase-like gene (*HOGA1*).

In veterinary medicine, PH1 has been described in dogs (OMIA 001672-9615) with a breed-specific homozygous missense variant in *AGXT* (XP_003639939.1:p.Gly102Ser) and identified as a cause in the Coton de Tulear breed [2]. PH2 has been reported in cats (OMIA 000821-9685) with a splice site variant in *GRHPR* found in the affected kittens [3]. In cattle, rare cases of neonatal oxalate nephropathy in purebred calves with no known exposure to exogenous oxalates were reported [4,5]. In Zwartbles sheep, severe oxalate nephropathy was previously described with suspicion of hereditary predisposition on the basis of epidemiological findings in Great Britain and Northern Ireland [6,7]. The observed age of onset was quite variable (three weeks to three years) but the clinical signs and histopathology were similar in all the studied cases [6]. The prevalence of PH in sheep has not been studied prospectively, but data from the 2002–2009 period suggested that the incidence of all forms of urinary system disease in Zwartbles sheep is higher (7.2%, 13/179 submissions) compared to all other breeds (0.86%, 909/105,176 submissions) [6]. Additionally, our own data from ovine diagnostic submissions to SRUC Veterinary services during the period January 2015–end of August 2020 indicated that lesions of PH were detected only in pedigree Zwartbles sheep, comprising 6.5% (3/46 submissions) of purebred Zwartbles sheep.

The aim of this study is to describe the phenotype and to identify the underlying genetic variant in several purebred Zwartbles sheep affected by a recessively inherited metabolic disease. Herein, we present evidence that an ovine type I primary hyperoxaluria is due to a missense variant in the *AGXT* gene.

## 2. Materials and Methods

### 2.1. Ethics Statement

This study did not require official or institutional ethical approval as ‘non-experimental clinical veterinary practices’ are specifically excluded from being considered regulated procedures under The Animals (Scientific Procedures) Act, 1986, Section 2(8) as well as the Directive 2010/63/EU on the protection of animals used for scientific purposes, 2010, Article 1(5). All animals in this study were examined with the consent of their owners. All samples were obtained at postmortem examination of affected animals that had died and were submitted by the owner for laboratory diagnostic investigation.

### 2.2. Animals and DNA Samples

Tissue samples including kidney were collected for further analyses postmortem from nine purebred Zwartbles sheep suspected to be affected by primary hyperoxaluria in Scotland (*n* = 3), northern England (*n* = 1), and the Netherlands (*n* = 5) (Supplementary Table S1). Genomic DNA was isolated from the samples using the standard protocols of Maxwell RSC DNA Tissue and FFPE kits (Promega, Dübendorf, Switzerland).

### 2.3. Histopathology

Samples of kidneys from all Zwartbles sheep were fixed in 10% neutral buffered formalin and routinely processed to paraffin wax. Histopathological analyses were undertaken on hematoxylin and eosin-stained 5 µm sections. The characteristics of the crystals present were assessed also by viewing the sections with polarized light.

### 2.4. Whole-Genome Sequencing

In order to identify the causative variant, a whole-genome sequence (WGS) was generated after the preparation of a PCR-free fragment library for two PH1-affected sheep (case 1 and case 2). The sequence data mapping to the ovine reference genome assembly Oar_rambouillet_v1.0 and the calling of single nucleotide and small indel variants including the prediction of functional effects were described previously [8]. PLINK v1.9 software [9] was used for homozygosity mapping of possible disease-associated intervals shared by both cases. The overlapping homozygous regions were determined by the option --homozyg group while allowing four heterozygous markers per window in order to account for possible calling errors as suggested by Ceballos et al. [10]. Private protein-changing variants shared by the two PH1-affected animals were identified by comparison with 79 publicly available control genomes, including 21 domestic sheep breeds unrelated to Zwartbles as well as two wild sheep subspecies (Supplementary Table S2). In addition, the Sheep Genomes Project Variant Database of further 453 samples of 54 other sheep breeds [11] available from the European Nucleotide Archive (ENA) was searched for the presence of the identified variants. The integrative genomics viewer (IGV) software [12] was used for visual inspection and screening for structural variants.

### 2.5. Candidate Variant Validation

Polymerase chain reaction (PCR) and Sanger sequencing were used to validate and genotype the variant identified from the WGS results. Primers were designed using Primer-BLAST [13] and PCR products from genomic DNA were amplified using AmpliTaqGold360 MasterMix (Thermo Fisher Scientific, Waltham, MA, USA). The purified PCR amplicons were directly sequenced on an ABI3730 capillary sequencer (Thermo Fisher Scientific). The *AGXT* missense variant (XM_027966918.1: c.584G>A) was genotyped using the following primers: GCTCACCTGTGGGTATGGG (forward) and ACAAGCCAGTGCTCCTGTTC (reverse). The obtained sequences were analyzed using Sequencher 5.1 software (GeneCodes, Ann Arbor, MI, USA).

### 2.6. Protein Predictions

PROVEAN [14] and MutPred2 [15], in silico prediction tools, were used to predict the biological consequences of the discovered variants on the protein. The default scores of ≤2.5 and ≥0.68 were considered to predict a variant as deleterious by PROVEAN and MutPred2, respectively. All references to the ovine *AGXT* gene correspond to the accessions NC_040252.1 (NCBI accession), XM_027966918.1 (mRNA), and XP_027822719.1 (protein). The genome aggregation database (gnomAD) v2.1.1 [16] was searched for the corresponding variant in the human AGXT.

### 2.7. Availability of Data and Material

The whole-genome data has been made freely available at the ENA under study accession number PRJEB30931 (sample accessions SAMEA6531513, SAMEA6531514). All accession numbers of the used genomes are available in Supplementary Table S2. The sheep genomes project variant database of further 453 samples is deposited in ENA under accession number PRJEB14685.

## 3. Results

### 3.1. Clinical Findings

Several purebred Zwartbles sheep of both sexes were submitted for laboratory investigation with a suspicion of breed-specific oxalate nephropathy in Scotland (*n* = 3), northern England (*n* = 1), and the Netherlands (*n* = 5) (Supplementary Table S1).

Two 1–2-month-old lambs (case 1 and case 2) that died suddenly were submitted for laboratory investigation from a small flock in Scotland. The first had a history of intermittent dullness while the second had been unwell for several days with coughing noted by the owner. Two other lambs of similar age from this flock died but were not further investigated. Case 3 was a 10-day-old lamb originating from a different small flock that appeared stiff and then fitted and died. Two 3–4 week-old Zwartbles lambs had previously died suddenly on the same farm but were not submitted for further investigation.

A 4-year old Zwartbles ewe (case 4) from a farm in northern England was presented with progressive condition loss and malaise over about three weeks, followed by death. The owner noticed it to be often near the water trough, suggesting polydipsia. Biochemical analysis of a urine sample from case 4 revealed an increased result of 84 µmol/mmol oxalate:creatinine ratio. This is probably higher than background levels in sheep [17], which suggested a defect in oxalate metabolism in this animal.

Five more Zwartbles sheep from the Netherlands were submitted for postmortem investigation with suspected hereditary nephropathy because of the known purebred pedigree. A 3-month old lamb (case 5) showed retarded growth without improvement on antibiotics treatment, and 1.5 week of increasing weakness followed by death. A 4-month old lamb (case 6) died suddenly, with no previous health problems reported in the flock. A 2-month old lamb (case 7) died after a period of shortness of breath and fever with initial improvement on antibiotics. A 1-month old lamb (case 8) suddenly died and was sent in by the owner because five more lambs on the same farm had succumbed without prior clinical signs. Additionally, a 2.5-month old lamb (case 9) suddenly died without further health history. More details about each animal are reported in Supplementary Table S1.

### 3.2. Macroscopic and Histopathological Findings

**Figure 1:**
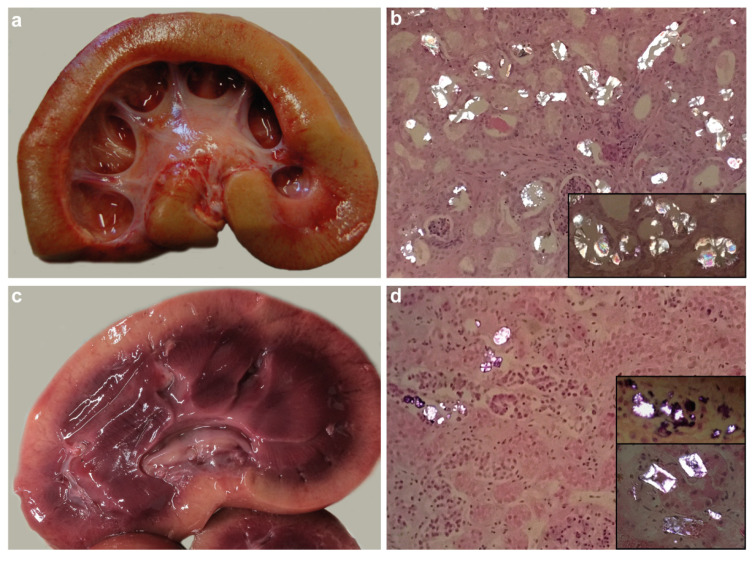
Macroscopic and histopathological findings in two Zwartbles sheep. (**a**) Kidney of case 3 showing the near-complete loss of corticomedullary demarcation and prominent dilation of the pelvis. (**b**) Numerous crystals in the renal tubules of case 3 are brightly birefringent when viewed by polarized light; the morphology is typical of oxalate (inset). (**c**) Kidney of case 4 showing cortical pallor and clear corticomedullary demarcation. (**d**) Sparse birefringent crystals in the renal tubules of case 4; the morphology is variable with some not typical of oxalate crystals (inset).

Postmortem examination of the examined sheep revealed moderate to good body condition and a urine-like smell (Supplementary Table S1). Kidneys of seven cases had pale firm cortices with a varying loss of cortico-medullary demarcation and prominent dilation of calyces and pelvices (Figure 1a). Histopathological analysis revealed severe chronic tubulointerstitial crystalline nephropathy with numerous intralesional oxalate crystals (Figure 1b) in most of the affected sheep (cases 1, 2, 3, 5, 6, 7, and 8). Limited purulent pyelonephritis associated with extension from omphalitis was also present in case 3. Figure 1 shows representative images of one case (case 3) in comparison with case 4. The kidneys of case 4 had pale cortices with clear demarcation from the purple medulla, with variable patency of renal calyces (Figure 1c). Histopathology of case 4 showed dilated tubules containing necrotic cell debris and purple-grey-black granular to crystalline deposits (Figure 1d). There was no evidence of end-stage renal disease, and the crystals were much less frequent than in the other cases with crystalline nephropathy. Case 9 had a purulent tubular and pyelonephritis with intralesional bacteria, *Escherichia coli* was cultured from the lesion.

Finally, on the basis of the comparison of clinicopathological findings, severe chronic fibrosing tubulointerstitial crystalline nephropathy with extensive oxalate crystal deposition was diagnosed in all but two cases (case 4 and case 9). In case 9, severe pyelonephritis due to *E. coli* has been found and no oxalate crystals were seen by histology, therefore disproving the initially suspected hyperoxaluria.

### 3.3. Identification of the Causative Variant

**Figure 2:**
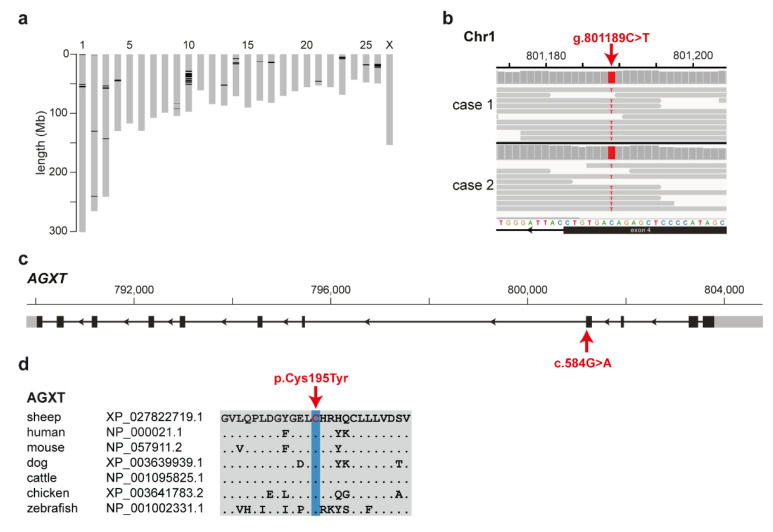
A missense variant in the alanine-glyoxylate aminotransferase (*AGXT*) gene is associated with type I primary hyperoxaluria (PH1) in Zwartbles sheep. (**a**) Representation of ovine chromosomes (grey bars) with highlighted regions of shared homozygosity (in black) in the two PH1-affected sheep with whole-genome sequence (WGS) data. (**b**) IGV [12] screenshot of the PH1-affected sheep WGS shows the missense variant present in both cases. (**c**) Schematic representation of the *AGXT* gene showing the variant location in exon 4. (**d**) Conservation of the affected amino acid in the AGXT protein across multiple species.

On the basis of the purebred pedigree history of the analyzed sheep and the recessive mode of inheritance described in other species for PH1, we hypothesized that a rare breed-specific deleterious variant is responsible for the described phenotype in the Zwartbles breed. Homozygosity mapping was used to identify intervals of extended homozygosity shared across both cases with available WGS (case 1 and case 2). This revealed 40 genomic regions representing 1.7% of the ovine reference sequence (Figure 2a). Visual inspection of the regions in the WGS of the affected sheep revealed no obvious structural variants. Filtering of the WGS data yielded 2,486 homozygous variants shared by both PH1-affected Zwartbles sheep and absent from the 79 controls (Supplementary Table S3). Out of those, 23 variants were protein-coding and only 10 of them were located within the detected homozygous intervals (Table 1). Beside three synonymous, seven missense variants in seven genes were found (Table 1). Two variants were also present heterozygously with low frequency in an independent control cohort of 453 genomes of unrelated sheep breeds [11]. Finally, only two variants in *ERICH3* and *AGXT* were predicted deleterious of which the *ERICH3* variant occurred rarely in other breeds (Table 1).

Moreover, the Zwartbles-specific missense variant in the *AGXT* gene (Figure 2b) was predicted as deleterious by both prediction tools (Table 1) and was subsequently pursued as a functional candidate, due to the gene’s previous involvement in human primary hyperoxaluria [18]. The ovine variant (chr1: g.801189C>T; c.584G>A; p.Cys195Tyr) is located in exon 4 of the *AGXT* gene (Figure 2c) and affects a highly conserved amino acid residue within the large N-terminal domain of the AGXT protein (Figure 2d). MutPred2 [15] also predicted the probability (Pr) of the *AGXT* variant’s impact on the following molecular mechanisms: altered metal binding (Pr = 0.72, *p*-value = 0.0009), gain of relative solvent accessibility (Pr = 0.30, *p*-value = 0.008), loss of allosteric site at H196 (Pr = 0.20, *p*-value = 0.04), altered transmembrane protein (Pr = 0.10, *p*-value = 0.04), and loss of pyrrolidone carboxylic acid at Q199 (Pr = 0.06, *p*-value = 0.03).

A different heterozygous missense variant (p.Cys173Trp, rs180177232) at the corresponding position of the human AGXT transcript was found in gnomAD [16] in a single genome. Further literature search revealed that a pathogenic missense variant with the same amino acid exchange in humans (p.Cys173Tyr, rs180177231) was associated with severely decreased catalytic activity and negative immunoreactivity in vitro and was found heterozygous in one PH1 patient [19]. Recently, another variant at the same position (p.Cys173Arg) was described in two closely related human PH1 patients exhibiting compound heterozygosity [20].

### 3.4. Targeted Genotyping

To experimentally confirm the *AGXT* missense variant (NC_040252.1: g.801189C>T), all nine available Zwartbles sheep were genotyped using Sanger sequencing. All seven PH1-affected sheep were homozygous for the variant (Supplementary Table S1). Case 4 was a heterozygous carrier and case 9, diagnosed with pyelonephritis, was homozygous for the wild type allele.

## 4. Discussion

On the basis of the presented clinicopathological data, an inherited form of type I primary hyperoxaluria characterized by severe chronic crystalline (oxalate) nephropathy was diagnosed in seven Zwartbles sheep homozygous for the *AGXT* missense variant. In contrast, an adult Zwartbles sheep with tubular necrosis and low grade tubular crystal formation was heterozygous for the variant. A ninth Zwartbles lamb that was suspected to have the disease was shown to have a bacterial pyelonephritis on postmortem examination and did not carry the identified variant. A WGS-based precision medicine approach was used to identify the underlying genetic variant in *AGXT* responsible for the described phenotype.

PH1 is caused by a loss of activity of the liver peroxisomal enzyme, which leads to a formation of insoluble calcium oxalate crystals. In human patients, the disease has a heterogeneous clinical phenotype with a very variable age of onset (early infancy to the 6th decade) as well as a severity of the disease [21]. Even within one family, the presentation may vary from infantile renal failure to occasional stone formation and a mild-to-moderate reduction in kidney function in adults. However, all patients are at an increased risk of developing end-stage renal disease eventually leading to death [21]. Molecular genetic testing is commonly used to identify pathogenic variants in the *AGXT* gene, an obvious functional candidate.

There are about 200 variants described in the human *AGXT* gene and the causative mutations were found in more than 99% of patients. The majority of pathological mutations for human PH1 are single nucleotide changes [18]. The missense mutations primarily affect the folding of the AGXT protein, which leads to its decreased stability [19]. A mouse model of PH1 showed that the *Agxt*-null mice, despite almost normal histology, develop hyperoxaluria and crystalluria with males having a higher concentration of urinary oxalate than females [22].

The interstitial lesions in all three Scottish cases and four Dutch cases with crystalline nephropathy indicated that the oxalate crystal deposition was associated with a chronic progressive renal disease similar to that reported in human PH1 [23]. The WGS of two PH1-affected sheep were used to map the disease-associated locus and to identify the most likely pathogenic *AGXT* variant (p.Cys195Tyr), which was absent from 532 unrelated control sheep. This amino acid exchange affects a residue highly conserved across multiple species and the ovine position Cys195 corresponds to amino acid position Cys173 of human AGXT protein. Several mutations affecting this residue have been previously reported in humans affected by PH1 [18,20,24]. Most recently, two missense heterozygous variants (AGXT: p.Cys173Arg; p.Ser223Arg) were identified in two patients from one family [20]. Interestingly, while the two probands suffering from a severe infantile form of PH1 carried both variants, all four heterozygous carriers of only the p.Cys173Arg variant were affected by kidney stones. On the other hand, five heterozygous carriers of only the p.Ser223Arg variant showed no signs of disease [20]. Furthermore, a pathogenic p.Cys173Tyr variant showed severely decreased catalytic activity and negative immunoreactivity in vitro [19]. PH1 likely remains underdiagnosed because of the wide variability in its clinical presentation as well as patients with only one heterozygous variant found [21,24].

While seven of the nine Zwartbles sheep available in this study were genotyped homozygous for the described AGXT:p.Cys195Tyr variant, a single sheep was a heterozygous carrier. Even though both renal injury and crystal formation were present in this case, the oxalate crystal accumulation was relatively minor and might have been a result of renal failure rather than a cause. However, further research is needed to investigate if heterozygotes for *AGXT* variants are possibly more prone to oxalate deposition in a range of conditions. Detection of greater than background levels of oxalate crystals, even if not associated with end-stage renal pathology, might raise suspicion of carrier status, and this might potentially predispose to development of oxalate nephrosis on high oxalate diets. Lastly, one initially suspected case of oxalate nephrosis, but finally diagnosed with kidney inflammation due to a bacterial infection, was genotyped homozygous wild type. This example shows the difficulties of a precise phenotypic diagnosis based on signalment and clinical examination alone.

## 5. Conclusions

In conclusion, we identified a non-synonymous variant in a highly plausible functional candidate gene through WGS data analyses. Our results combined with the current knowledge on AGXT function in other species provide strong evidence for a breed-specific missense variant affecting a conserved residue of AGXT as the most likely causative genetic variant for recessively inherited type 1 primary hyperoxaluria in Zwartbles sheep. This is the first report of the underlying pathogenesis of PH1 in sheep that supports the efficiency of the chosen method in rare metabolic disease gene discovery and enables the development of a genetic test for veterinary diagnostic and breeding purposes. Identification of this variant should bring about improvements in animal welfare by enabling the screening of breeding animals to determine and reduce the prevalence of the PH1 in the Zwartbles sheep population.

## Figures and Tables

**Table 1:** Private protein-coding variants detected in the shared homozygous regions from whole-genome sequence (WGS) of two type I primary hyperoxaluria-affected Zwartbles sheep.

Variant Position ^1^	Gene	Protein Change	Allele Frequency ^2^	PROVEAN Score ^3^	MutPred2 Score ^4^
chr1:652874	*SNED1*	p.Glu747Lys	0	−1.279	0.628
chr1:801189	*AGXT*	p.Cys195Tyr	0	−9.768	0.891
chr1:54671486	*ERICH3*	p.Gly23Glu	0.0099	−4.526	0.852
chr10:36256345	*SPATA13*	p.Asp1073=	0.0036	NA	NA
chr10:38336210	*MPHOSPH8*	p.Arg426Gln	0	−0.638	0.085
chr14:14488283	*ZNF469*	p.Glu2351Lys	0	−1.756	0.271
chr17:13778922	*ZNF827*	p.Asn694Ser	0	−0.462	0.098
chr26:16667329	*CCDC110*	p.Asn649=	0	NA	NA
chr26:16820162	*SORBS2*	p.Pro394Leu	0	−0.301	0.121
chr26:17153454	*TLR3*	p.Leu244=	0	NA	NA

^1^ All positions refer to the Oar_rambouillet_v1.0 reference sequence assembly. Additional descriptive details are given in full in Supplementary Table S3. ^2^ The variant allele frequency detected in the 453 sheep Genomes Project Variant Database [11]. ^3^ PROVEAN score ≤–2.5 predicts a variant as deleterious [14]. ^4^ MutPred2 score ≥0.68 predicts a variant as deleterious [15].

## Supplementary Materials

The following are available online at https://www.mdpi.com/2073-4425/11/10/1147/s1, Table S1: Details of the investigated Zwartbles sheep, Table S2: Sample designations and detailed information of all whole-genome sequences, Table S3: Shared private variants in two Zwartbles sheep affected by type 1 primary hyperoxaluria and absent from 79 unrelated sheep genomes.
